# GMCL1 Controls 53BP1 Stability and Modulates Paclitaxel Sensitivity in Cancer

**DOI:** 10.1101/2025.03.18.643855

**Published:** 2025-03-19

**Authors:** Yuki Kito, Tania J. Gonza lez-Robles, Sharon Kaisari, Juhee Pae, Sheena Faye Garcia, Juliana Ortiz-Pacheco, Beatrix Ueberheide, Ruth Lehmann, Antonio Marzio, Gergely Rona, Michele Pagano

**Affiliations:** 1 Department of Biochemistry and Molecular Pharmacology, New York University Grossman School of Medicine, New York, NY 10016, USA; 2 Laura and Isaac Perlmutter Cancer Center, New York University Grossman School of Medicine, New York, NY 10016, USA; 3 Division of Precision Medicine, Department of Medicine, NYU Grossman School of Medicine, New York, NY 10016, USA; 4 Howard Hughes Medical Institute, NYU Grossman School of Medicine, New York, NY 10016, USA; 5 Department of Cell Biology, New York University Grossman School of Medicine, New York, NY 10016, USA; 6 Proteomics Laboratory, Division of Advanced Research Technologies, New York University Grossman School of Medicine, New York, NY 10016, USA; 7 Institute of Molecular Life Sciences, Research Centre for Natural Sciences, Budapest, 1117, Hungary.; 9 Lead contact

**Keywords:** GMCL1, prolonged mitosis, 53BP1, p53, mitotic stopwatch, protein degradation, ubiquitin

## Abstract

The Mitotic Surveillance Pathway (MSP) monitors the duration of M-phase. Prolonged mitosis, caused by spindle attachment defects or microtubule-targeting drugs such as the taxane paclitaxel, induces the formation of the ternary “mitotic stopwatch” complex consisting of 53BP1, USP28, and p53. This event protects p53 from degradation, resulting in cell cycle arrest or apoptosis in daughter cells. In paclitaxel-resistant cancers, cells bypass the MSP, enabling unchecked proliferation and survival, although the underlying mechanisms remain unknown. Here, we demonstrate that 53BP1 physically interacts with GMCL1 but not its paralog, GMCL2, and we mapped the interaction regions on both proteins. CRL3^GMCL1^ functions as a ubiquitin ligase that targets 53BP1 for degradation during M phase, impacting p53 levels in daughter cells. High GMCL1 expression significantly correlates with resistance to paclitaxel in cancer cell lines with wild-type p53, including endometrial, breast, and upper aerodigestive tract cancer cells. Loss of GMCL1 restores paclitaxel sensitivity in p53 expressing cells but not in p53 deficient cells. We propose that in cancers with high GMCL1 levels, the CRL3^GMCL1^-mediated degradation of 53BP1 prevents the formation of the mitotic stopwatch complex, leading to p53 degradation and sustained proliferation. Finally, our results indicate that GMCL1 inhibition represents a novel strategy to restore taxane sensitivity in resistant cancers.

## INTRODUCTION

Mitosis is orchestrated by several intracellular signaling pathways to ensure proper cell division while maintaining genomic integrity. Errors during cell division, including chromosome mis-segregation or spindle defects, could lead to changes in chromosome content, producing aneuploid or polyploid progeny cells, which could be detrimental during development or lead to oncogenesis^1,2^. Therefore, cells have evolved quality control mechanisms to ensure proper cell division during M phase. One such surveillance mechanism is known as the Mitotic Surveillance Pathway (MSP), which senses prolonged mitosis as an indicator of mitotic defects^3^.

If a threshold mitotic duration is surpassed, the MSP induces cell cycle arrest in the daughter cells in a p53-dependent manner^4–6^. During abnormally prolonged mitosis^7–10^, the MSP is triggered by the formation of a ternary “mitotic stopwatch” complex consisting of the deubiquitinase USP28 (ubiquitin-specific protease 28), 53BP1 (p53-binding protein 1), which acts as a substrate recognition subunit, and p53. During delayed mitosis, formation of the mitotic stopwatch complex allows USP28 to deubiquitinate and subsequently protect p53 from MDM2-mediated degradation. The 53BP1-USP28-p53 complex accumulates and persists in the G1 phase of daughter cells, where it promotes cell cycle arrest *via* p53-mediated upregulation of p21 expression.

Notably, proper mitotic arrest plays a crucial role in the mechanism of microtubule-targeting therapies, such as taxanes (e.g., paclitaxel and docetaxel), which disrupt spindle formation and chromosomal segregation. Recent studies have shown that pharmacological interventions targeting M phase, even without resulting in complete mitotic arrest, can induce controlled cell death^11^. For example, low dose paclitaxel treatments results in increased p21 expression and apoptosis in daughter cells^12^. However, there are cancers that display resistance or insensitivity to treatment with taxanes, including metastatic breast cancer, ovarian cancer, and non-small cell lung cancer^13–15^. Therefore, there is an urgent need to elucidate the strategies of paclitaxel resistance in such cancers.

Human germ cell-less protein-like 1 (GMCL1) is a putative substrate receptor of one of many CUL3-RING ubiquitin ligases (CRL3s). However, to date, GMCL1’s biological role and its substrates have remained uncharacterized. *GMCL1* received its namesake from its *Drosophila melanogaster* homolog, *GCL* (Germ Cell-Less), which plays essential roles in early embryonic development and germ cell determination^16,17^. *GMCL1*’s role in germ cell development appears to have been evolutionarily conserved as loss of *GMCL1* expression in mice has been shown to cause defects in meiosis and spermiogenesis^18^, and altered *GMCL1* expression was functionally associated with human asthenozoospermia^19^. Although *GMCL1* homologs have been primarily associated with germ cell biology, several databases (e.g., GTEx^20^ and GENT2^21^) indicate that *GMCL1* is expressed in somatic cells as well, suggesting broader biological functions beyond germ cell development. In contrast, the GMCL1 paralog GMCL2 is specifically expressed in germ cells.

Here, we present studies that shed light on the role of GMCL1 in somatic cells. We demonstrate that, like its *D. melanogaster* counterpart, GMCL1 acts as a CRL3 substrate receptor. We also identify 53BP1 as a *bona fide* substrate of CRL3^GMCL1^, demonstrating that its levels are regulated by GMCL1 during M phase. Reduction of mitotic 53BP1 by GMCL1 prevents the formation of the USP28-p53–53BP1 mitotic stopwatch complex and reduces p53 transmission to daughter cells. We therefore propose that GMCL1 inhibition, in combination with paclitaxel therapy, offers a promising strategy to overcome taxane resistance.

## RESULTS

### Identification of 53BP1 as an interactor of CRL3^GMCL1^

We have shown that the GMCL1 ortholog in *D. melanogaster*, GCL, is a substrate recognition subunit of a CRL3 complex that is active specifically in mitosis^22^. So, we predicted the human GMCL1 to also behave as a CRL3 substrate receptor. GMCL1 contains a BTB (Broad-Complex, Tramtrack, and Bric-à-brac) and a BACK (BTB and C-terminal Kelch) domain (Supplementary Figure 1A), consistent with other CRL3 receptors. On its C-terminus, GMCL1 also contains a GCL domain (residues 379–515), which is distinct from the Kelch domains commonly used by other CUL3 substrate receptors since it is predicted to form a β-sandwich characterized by two opposing anti-parallel β-sheets, each made up of four β-strands^22,23^ (Supplementary Figure 1A). A Dali search^24^ of GMCL1’s C-terminal domain reveals that it has some structural homology to the MATH (meprin and TRAF homology) domain used by another CRL3 substrate receptor, SPOP^25^, suggesting that the predicted structure of GMCL1’s GCL domain could also potentially act as a protein-protein interaction motif to recruit substrates.

**Figure 1.**
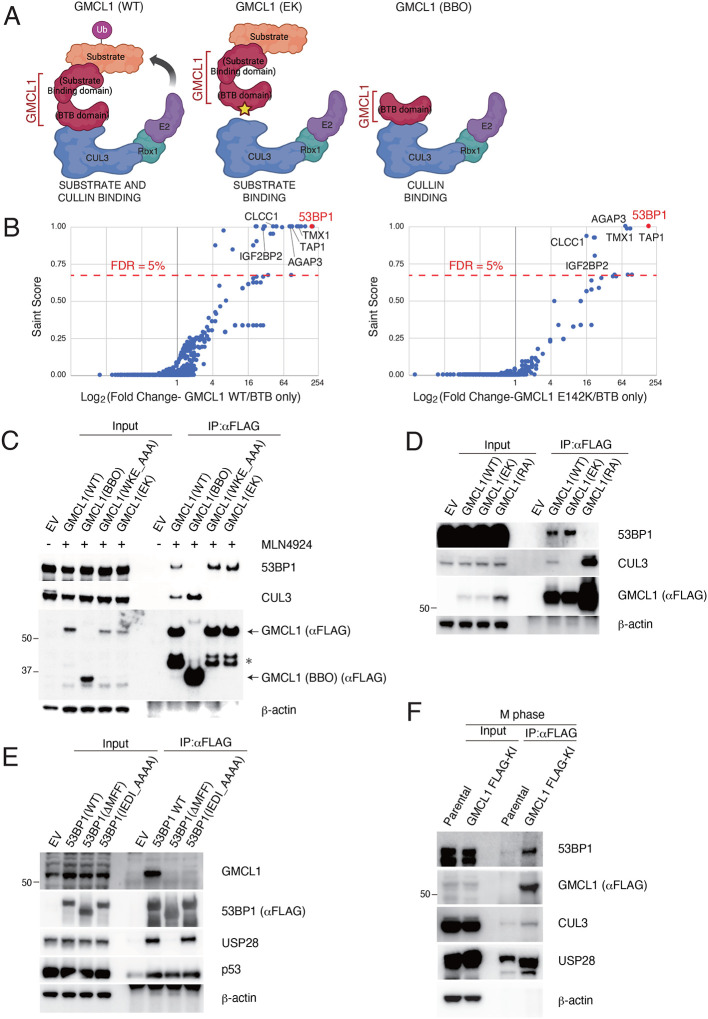
Identification of 53BP1 as a GMCL1 interactor A) Schematics for the immunoprecipitation-mass spectrometry (IP-MS) workflow using wild-type GMCL1 (GMCL1 WT) and mutants (GMCL1 EK and GMCL1 BBO). Color coding: Red, GMCL1; orange, putative substrates/interacting partners; blue, CUL3; green, RBX1; purple, E2 ubiquitin-conjugating enzyme. B) HEK293T cells were transfected with FLAG-GMCL1 WT, FLAG-GMCL1 EK, or FLAG-GMCL1 BBO. After 24 hours, FLAG-tagged proteins were immunoprecipitated and analyzed by MS/MS. Left panel: proteins enriched with GMCL1 WT vs. BBO; right panel: proteins enriched withGMCL1 EK vs. BBO. Significant interactors were identified using SAINT scores > 0.70 and FDR < 5%. C) HEK293T cells transfected with empty vector (EV), FLAG-GMCL1 WT, FLAG-GMCL1 BBO, FLAG-GMCL1 WKE_AAA (broadly disrupts the binding to CUL3) and FLAG-GMCL1 EK were treated with MLN4924 (3h). 53BP1 and CUL3 were immunoprecipitated with FLAG beads and analyzed by western blot. Asterisk indicates non-specific bands. D) HEK293T cells were transfected with EV, FLAG-GMCL1 WT, FLAG-GMCL1 EK, or FLAG-GMCL1 RA. FLAG immunoprecipitations were probed for 53BP1 and CUL3. E) HEK293T cells were transfected with EV, FLAG-53BP1 WT, FLAG-53BP1 ΔMFF and FLAG-53BP1 IEDI_AAAA. After MLN4924 treatment (3h), GMCL1 was immunoprecipitated and immunoblotted. F) M phase-synchronized GMCL1 FLAG knock-in HCT116 cells were collected. GMCL1 was immunoprecipitated using FLAG-beads and analyzed by immunoblotting.

To investigate the role of GMCL1 in somatic cells, we used immunoprecipitation followed by mass spectrometry (IP-MS) to identify binding partners of GMCL1. Proteomics studies were performed by expressing and purifying the following FLAG-tagged proteins: (*i*) wild-type GMCL1 (GMCL1 WT), (*ii*) GMCL1 E142K (GMCL1 EK), which carries a mutation in the BTB domain that is predicted to disrupt its interaction with CUL3, and (*iii*) GMCL1 BTB/BACK-only (GMCL1 BBO) that lacks the GCL domain (Figure 1A–C). IP-MS analysis identified 1,765 potential binding partners that specifically interact with GMCL1 *via* its C-terminal domain. Using SAINT scores > 0.70 and FDR < 5%, this list was refined to 9 proteins that showed significant interaction with GMCL1 WT and GMCL1 EK, but not with GMCL1 BBO, with 53BP1 being the one of most enriched protein (Figure 1B, **Supplementary Table 1**). This selective enrichment suggests that the C-terminal, “MATH-like” GCL domain of GMCL1 is critical for its interaction with binding partners, including 53BP1. The interaction between GMCL1 and endogenous 53BP1 and CUL3 was validated using immunoprecipitations (IPs) followed by immunoblotting (Figure 1C,D).

To further study the direct interaction between GMCL1 and 53BP1, we used AlphaFold 3^26^ to predict their binding interface and identify potential critical residues at the recognition interface between the substrate receptor and 53BP1to see if mutating these residues could abolish their interaction. Consistent with our initial IP-MS experiment, the Alphafold 3 model predicted that the C-terminal domain of GMCL1 would interact with 53BP1. Based on the GMCL1–53BP1 complex structure prediction, we identified Arg 433, which appears to be a solvent-exposed residue in GMCL1’s GCL domain that could interact with 53BP1 without impeding GMCL1’s binding to with CUL3. Thus, we generated the GMCL1 R433A (GMCL1 RA) point mutant and tested its binding to 53BP1 using IP. As predicted, compared to WT GMCL1, the R433A mutation completely abolished the binding of GMCL1 to 53BP1, but did not impact GMCL1’s binding to CUL3 (Figure 1D).

To determine which region of 53BP1 mediates its binding to GMCL1, we mapped the predicted GMCL1-binding site on 53BP1 and identified a conserved IEDI amino acid sequence within the Minimal Focus Forming region (MFF) of 53BP1^27^ (Supplementary Figure 1B–E). Through IPs, we demonstrate that a 53BP1 mutant either lacking the MFF region or containing the IEDI-to-AAAA mutation within this region lost its interaction with CRL3^GMCL1^, suggesting that this conserved IEDI sequence within 53BP1’s MFF forms a critical degron recognized by GMCL1 (Figure 1E).

To examine the interaction between endogenous GMCL1 and endogenous 53BP1, we used CRISPR-Cas9 to knock in a FLAG tag at the C-terminus of *GMCL1*. Immunoprecipitation experiments confirmed that endogenous GMCL1 interacts with both endogenous CUL3 and 53BP1 (Figure 1F).

Finally, we sought to determine whether GMCL2, a GMCL1 paralog, is also able to interact with 53BP1. Immunoprecipitation of FLAG-tagged GMCL1 or GMCL2 from HEK293T cells revealed that while GMCL1 binds to 53BP1, GMCL2 does not (Supplementary Figure 1F), suggesting that GMCL1 and GMCL2 have distinct functions.

Overall, our results suggest that GMCL1 is a *bona fide* CRL3 substrate receptor and interacts specifically with 53BP1.

### GMCL1 loss leads to 53BP1 accumulations under prolonged M phase

**Figure 2.**
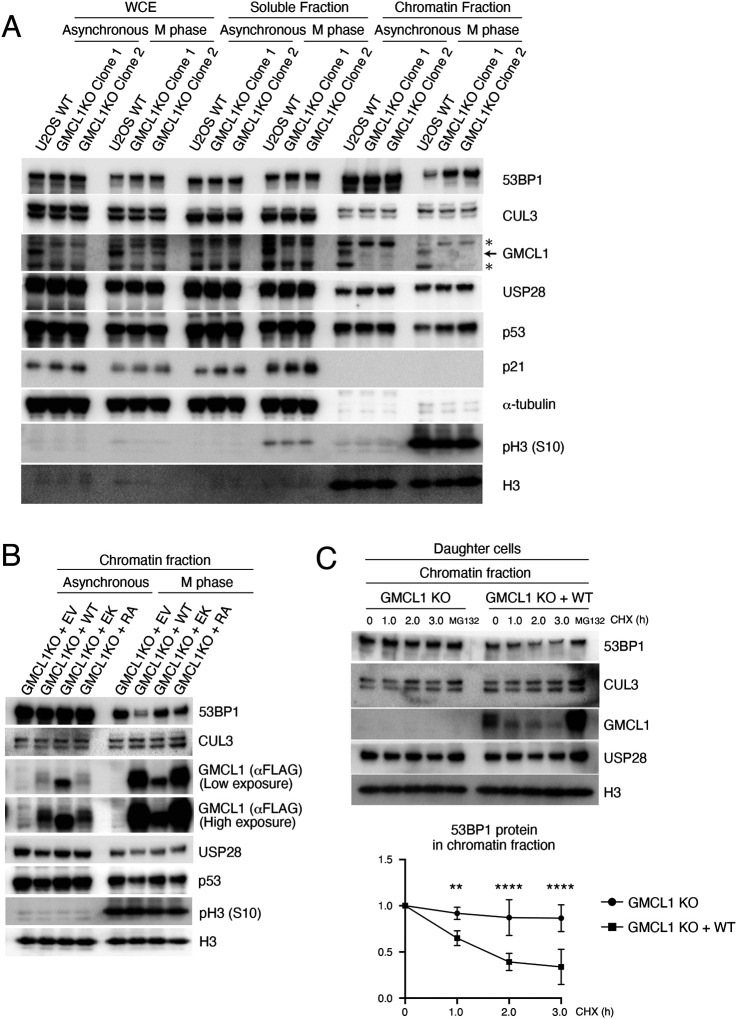
GMCL1 targets 53BP1 for degradation during M phase A) Asynchronous or M phase-synchronized WT or GMCL1 knockout (KO) U2OS cells were collected. Whole-cell extracts (WCE) were prepared using RIPA buffer, and other lysates were fractionated into soluble and chromatin-bound fractions for immunoblotting. Arrow indicates GMCL1-specific bands. Asterisk indicates non-specific bands. B) Stable U2OS cell lines expressing EV, FLAG-GMCL1 WT, FLAG-GMCL1 EK, or FLAG-GMCL1 RA in a GMCL1 KO background were synchronized into M phase and fractionated for chromatin immunoblotting. C) Mitotic-synchronized FLAG-GMCL1-expressing cells were collected by shake-off and cultured in fresh FBS-containing medium for 7 h. Daughter cells were treated with CHX at the indicated time points and collected within 7 hours, fractionated into soluble and chromatin-bound fractions, and analyzed by immunoblotting.

To investigate whether GMCL1 regulates 53BP1 stability, we generated *GMCL1* knock-out (KO) cells using CRISPR Cas-9^28^ and compared 53BP1 levels between *GMCL1* WT and two *GMCL1* KO clones. We found that 53BP1 levels were significantly increased in our *GMCL1* KO cells during M phase. While the whole cell extracts (WCE) showed modest differences in GMCL1 levels between the *GMCL1* WT and KO clones, our fractionation experiments revealed that 53BP1 mainly accumulated in the chromatin-bound fraction of *GMCL1* KO cells (Figure 2A).

To further explore the role of GMCL1 on 53BP1 stability, we stably reconstituted U2OS *GMCL1* KO cells with either GMCL1 WT or binding mutants (i.e., GMCL1 EK and GMCL1 RA). Notably, the accumulation of 53BP1 in *GMCL1* KO cells was rescued only upon re-expression of GMCL1 WT. In contrast, 53BP1 levels remained high in *GMCL1* KO cells expressing either GMCL1 EK or GMCL1 RA (Figure 2B), emphasizing the importance of GMCL1’s ability to bind both CUL3 (through the E142 residue) and 53BP1 (through the R433 residue) to regulate 53BP1 levels. Furthermore, GMCL1 WT re-expression in *GMCL1* KO cells led to decreased mRNA levels of p21 and NOXA during mitosis, whereas the GMCL1 EK and GMCL1 RA mutants failed to suppress these transcripts (Supplementary Figure 2A).

Collectively, these findings indicate that GMCL1 modulates 53BP1 availability through interactions with the CRL3^GMCL1^ complex, playing a critical role in the regulation of 53BP1 levels in M phase.

During mitosis, 53BP1 has been implicated in monitoring centrosome integrity and mitotic progression *via* the Mitotic Stopwatch Pathway (MSP). A recent study demonstrated that cells experiencing abnormally prolonged mitosis transmit this information to daughter cells though a ternary complex containing 53BP1, USP28, and p53 called the “mitotic stopwatch” complex^7^.

To investigate whether GMCL1 regulates this complex, we analyzed GMCL1 levels in post-mitotic daughter cells seven hours after release from prolonged nocodazole arrest. *GMCL1* KO daughter cells, reconstituted with GMCL1 WT, successfully re-entered the cell cycle, exhibiting low levels of 53BP1, p53, and p21, along with reduced expression of apoptosis-related genes (Supplementary Figure 2B,C). In contrast, cells reconstituted with either the E142K or R433A mutant displayed persistently elevated levels of 53BP1, p53, and p21, accompanied by increased expression of apoptosis-related genes (Supplementary Figure 2B,C). Notably, compared to parental cells, GMCL1 KO cells, whether reconstituted with WT or mutant GMCL1 constructs, showed no significant alterations in cell cycle profiles (Supplementary Figure 2D).

Next, we used cycloheximide (CHX) to inhibit translation and to assess the stability of 53BP1. We observed that in the chromatin-bound fraction of *GMCL1* KO cells, 53BP1 was more stable compared to cells rescued with GMCL1 WT (Figure 2C).

These results suggest that cells lacking GMCL1 that experienced prolonged mitosis will maintain elevated levels of p53, increasing the likelihood of apoptosis in subsequent cell cycles.

### GMCL1 modulates paclitaxel resistance in cancer cells

**Figure 3.**
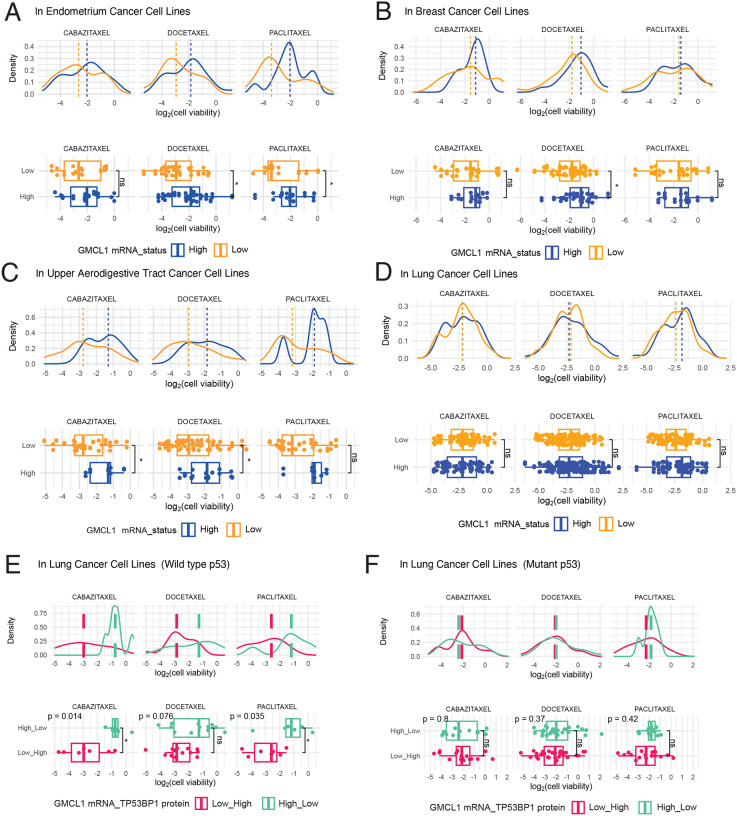
GMCL1 expression shows positive correlation with Taxol resistance in cancel cell lines For each cancer type (A-D) and p53 status (E-F), two visualization methods are presented: Upper panels: Density plots show the distribution of drug responses (log2[cell viability]) to the indicated taxane. Each curve represents the frequency of cell lines exhibiting specific response values. Blue curves represent high GMCL1 expression, while orange curves show low GMCL1 expression. Vertical dashed lines indicate median response values for each group. Rightward shifts of blue curves (high GMCL1) indicate greater resistance. Lower panels: Boxplots of the same data showing median (horizontal line), interquartile range (box), and distribution range (whiskers). Higher values on the y-axis indicate greater resistance to the drug. Statistical significance is indicated (*p < 0.05; ns: not significant) using Wilcoxon rank-sum test. In panels E-F, cell lines are stratified by both GMCL1 mRNA and 53BP1 protein levels as ‘Low_High’ (low GMCL1/high 53BP1, red) or ‘High_Low’ (high GMCL1/low 53BP1, green). Note the significant differences in panels E (wild-type p53) but not in panels F (mutant p53), demonstrating that GMCL1-mediated taxane resistance requires functional p53 signaling.

Paclitaxel, a widely used chemotherapeutic drug, stabilizes microtubules and prevents their depolymerization, thereby halting cancer cell division and inducing apoptosis. To assess the clinical significance of GMCL1, we investigated the relationship between GMCL1 expression levels and Taxol resistance in various cancer cell lines. To this end, we leveraged the PRISM (profiling relative inhibition simultaneously in mixtures) repurposing dataset, which compiles the proliferation inhibitory activity of 4,518 compounds tested across 578 patient-derived cancer cell lines^29^. This high-throughput screening dataset provides valuable insights into drug resistance mechanisms. We examined the impact of GMCL1 mRNA expression on Taxol resistance by analyzing taxane-related compounds from this dataset, including cabazitaxel, docetaxel, and paclitaxel, integrating it with the Cancer Dependency Map (https://depmap.org). Interestingly, we found that several cancer types (*e.g*.: endometrial, breast, and upper aerodigestive tract cancers) with high levels of GMCL1 mRNA exhibited resistance to paclitaxel, cabazitaxel, and/or docetaxel (Figure 3A–C). In contrast, lung cancer cell lines with high GMCL1 mRNA expression did not show such tendencies (Figure 3D). Given that nearly half of lung cancers harbor p53 mutations^30^, we hypothesized that p53 status may influence GMCL1-mediated Taxol resistance. To further investigate the functional relationship between GMCL1, 53BP1 and p53 in Taxol resistance, we stratified cancer cells based on their p53 status (wild-type vs mutant) and analyzed GMCL1 mRNA expression and 53BP1 protein levels. In cancer cells with wild type p53, high GMCL1 mRNA expression and low 53BP1 protein levels correlated with significant increased resistance to Cabazitaxel and Paclitaxel when compared to cells with low GMCL1 mRNA and high 53BP1 protein levels (Figure 3E). However, this effect was abolished in p53-mutant cells, where GMCL1 status no correlated with Taxol resistance (Figure 3F).

**Figure 4.**
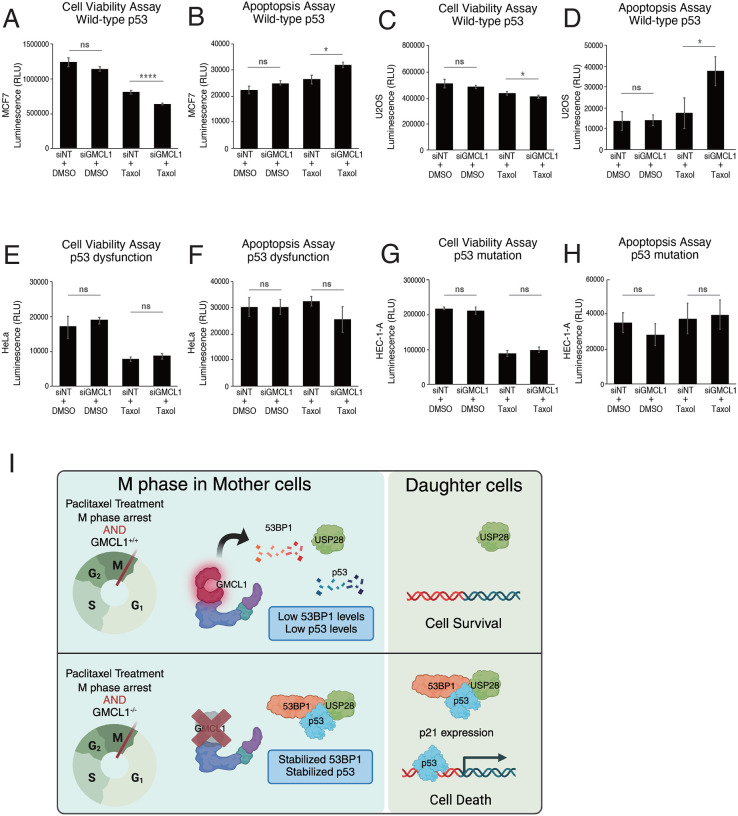
GMCL1 deficiency sensitizes cancers with wild-type p53 to Taxol-induced apoptosis MCF7 (A), U2OS (C), HeLa (E), HEC-1-A (G) cells were transfected with GMCL1-targeting siRNAs or non-targeting (NT) control for 72 h. Cells were treated with DMSO or Taxol for 48 h, and cell viability was assessed using the CellTiter-Glo Cell Viability Assay. Error bars represent standard deviation. Apoptosis was measured in the same conditions, i.e., MCF7 (B), U2OS (D), HeLa (F), HEC-1-A (H), using the RealTime-Glo Annexin V Apoptosis and Necrosis Assay. Error bars represent standard deviation. (I) Overview of GMCL1’s function during the M phase. In cancers with high GMCL1 levels, the CRL3^GMCL1^-mediated degradation of 53BP1 prevents the formation of the mitotic stopwatch complex, leading to p53 degradation and sustained proliferation. Loss of GMCL1 stabilizes mitotic 53BP1 levels and restores paclitaxel sensitivity.

To further verify the impact of GMCL1 levels on Taxol sensitivity, we performed cell viability and apoptosis assays using cells with wild type or mutant p53. In p53 wild-type cells (MCF7 and U-2OS), Taxol treatment led to a significant reduction in cell viability and an increase in apoptosis in GMCL1-depleted cells compared to cells transfected with non-targeting control siRNA (Figure 4A–D). However, GMCL1 knockdown did not affect cell viability or apoptosis in Taxol-treated cells with mutant p53 or inactivated p53 (HeLa and HEC-1-A, respectively) (Figure 4E–H).

These observations are consistent with the results shown in Figure 3 and support the hypothesis that Taxol resistance is mediated by GMCL1 through the USP28-p53–53BP1 complex. Specifically, high GMCL1 expression promotes the degradation of 53BP1, maintaining low p53 levels, thereby reducing Taxol-induced cell death in cell that have functional p53.

## DISCUSSION

We identify GMCL1, a previously uncharacterized human CRL3 substrate receptor, as a regulator of taxane resistance. Specifically, we found that GMCL1 interacts with and mediates the degradation of 53BP1 during prolonged arrest in M phase. While 53BP1 is well known for its role in double-strand break (DSB) repair via non-homologous end joining (NHEJ)^31^, it also participates to a so-called mitotic stopwatch composed of the 53BP1-USP28-p53 complex that stabilizes p53 during prolonged mitotic arrest^7–9^. We showed that GMCL1 controls the levels of 53BP1 and, consequently, those of p53 in mitotic cells, thereby influencing p53 transmission to daughter cells (see model in Figure 4I). This data suggests that GMCL1 functions as a mitotic stress regulator, with potential oncogenic properties in certain contexts.

Cancer database analysis reveals that GMCL1 is overexpressed in multiple cancers, including bladder, brain, breast, kidney, liver, lung, ovary, prostate, skin, and stomach cancers (Supplementary Figure 3). This underscores GMCL1’s role in mitotic regulation and genomic stability, which may vary based on tumor type, genetic background, and additional oncogenic mutations. Recent studies indicate that the MDM2-p53 axis^10^, along with the USP28-53BP1-p53 complex, functions as a mitotic timer enforcing G1 arrest following prolonged cell division. Our study identifies GMCL1 as an E3 ligase regulating this pathway by mediating 53BP1 degradation in mitosis. As often is the case for crucial regulatory proteins, 53BP1 is subject to regulation by multiple factors under different cellular circumstances^32–34^.

GMCL1 has been primarily studied in the germ cells of *D. melanogaster*, where we have shown that it forms an active CRL3 complex during mitosis. Our new findings establish a critical role for GMCL1 in somatic cell fate decisions by regulating 53BP1 stability during prolonged mitosis. We found that GMCL1 loss enhances Taxol-induced cell death in p53 wild-type cancers but has no effect in p53-mutant cells (Figure 4I). This suggests that GMCL1 inhibition could be selectively effective in cancers that retain functional p53. These findings position GMCL1 as a key modulator of mitotic stress and Taxol resistance, highlighting its potential as a therapeutic target in cancer treatment.

## MATERIALS AND METHODS

### Cell culture

Cell lines were purchased from ATCC and were routinely checked for mycoplasma contamination with the MycoStrip Mycoplasma Detection Kit (Invivogen). HEK293T (ATCC CRL-3216), HeLa (ATCC CCL-2) were maintained in Dulbecco’s modified Eagle’s medium (DMEM) (Gibco). U-2 OS (ATCC HTB-96), HCT-116 (ATCC CCL-247) and HEC-1-A (ATCC HTB-112.NM) cells were maintained in McCoy’s 5A medium (Gibco). MCF7 (ATCC HTB-22) were maintained in Eagle’s Minimum Essential Medium (EMEM) (ATCC). All media were supplemented with 10% fetal bovine serum (FBS) (Corning Life Sciences) and 1% penicillin/streptomycin/L-glutamine (Corning Life Sciences); however, MCF7 was further supplemented with 0.28% human recombinant insulin, zinc solution (11.2 μg/ml, Gibco). All cell lines were maintained at 37 °C and 5% CO2 in a humidified atmosphere.

### Plasmids, siRNA, and transfection

*Homo sapiens* cDNAs were amplified by PCR using KAPA HiFi DNA Polymerase (Kapa Biosystems) and sub-cloned into a variety of vector backbones, including modified pCDNA3.1 vectors containing C-terminal Flag tags, and pLVX-PURO lentiviral vectors containing C-terminal Flag. Site-directed mutagenesis was performed using KAPA HiFi DNA Polymerase (Kapa Biosystems). All cell lines were transiently transfected using Lipofectamine 3000 (ThermoFisher Scientific) based on the manufacturer’s recommendation. siRNA oligo transfections were performed using RNAiMax (ThermoFisher Scientific) according to the manufacturer’s instructions.

### Virus-mediated gene transfer

For the generation of lentivirus, HEK293T cells were transfected with pLVX constructs carrying the genes of interest, alongside the packaging plasmids pCMV-delta-R8.2 and pCMV-VSV-G. Viral supernatant was harvested 48 hours post-transfection, passed through a 0.45-μm sterile Millex-HV filter unit (Millipore Sigma), and supplemented with polybrene at a final concentration of 8 μg/ml (Sigma). Target cells were infected by replacing their culture medium with the virus-containing supernatant for an 8-hour incubation period. Selection of successfully transduced cells was performed using puromycin at a concentration of 1–2 μg/ml (Sigma).

### CRISPR-Cas9 genome editing

CRISPR–Cas9 genome editing techniques were carried out as previously described^35^ with modifications. In brief, to generate *GMCL1*-knockout U-2 OS cells, optimal gRNA target sequences closest to the start codon of the genes were designed using the Benchling CRISPR Genome Engineering tool (https://www.benchling.com). For transient Cas9 expression, gRNAs specific for *GMCL1* gene was incorporated into the pRP [CRISPR]-Hygro-hCas9-U6 vector, which was obtained from VectorBuilder (https://en.vectorbuilder.com/). The following oligos were used to generate the proper gRNA in the vector: GMCL1 (5’-CGTGCCCCCACGTACCTTCG-3’). To generate *GMCL1* 2×Flag knock-in HCT116 cells, an optimal gRNA target sequence closest to the genomic target site and a ~2 kb homologous recombination (HR) donor template was designed using the Benchling CRISPR Genome Engineering tool. The HR donor template was designed to introduce a 2×Flag tag in frame with the C terminus of *GMCL1*, in the following order: GMCL1-linker-FLAG-linker-FLAG-Stop codon and was purchased from VectorBuilder (https://en.vectorbuilder.com/). The following single gRNA sequence was used for the transient hCas9 expression vector: GMCL1 (5’-AAGTTACAGCAGATATATAA-3’).

Genomic DNA was collected using QuickExtract (Epicentre). Genotyping PCRs were performed with MyTaq HS Red Mix (Bioline), using primers surrounding the genomic target sites. The following primers were used for genotyping: GMCL1 (F: 5’-GCAGGCTTCTGATCTTCCCT-3’, R: 5’-ACTTGTCATCGTCGTCCTTGT-3’), and GMCL1 (F: 5’=GGGTGGGAGTTTGGAGAGTG-3’, R:5’-TCTGGATTTTCTGGGTGACGA-3’). The resulting PCR products were then purified and sequenced to determine the presence of insertion or deletion events. Clones positive to insertion or deletion events were then validated by western blot.

### Antibodies

The following antibodies were used: β-actin (1:5,000, Sigma-Aldrich A5441), CUL3 (1:1,000, Bethyl Laboratories A301–109A), FLAG (1:2,000, Sigma-Aldrich F7425), GMCL1 (1:1,000, Proteintech 15575–1-AP), HA (1:2,000, Bethyl Laboratories A190–108A), Histone H3 (1:1,0000, Abcam, ab1791), p21 (1:1,000, Cell Signaling Technology 2947S), p53 (1:1,000, Proteintech 10442–1-AP), pHistone H3 (D2C8) (Ser10, 1:1,000, Cell Signaling Technology, #3377), USP28 (1:1,000, Proteintech 17707–1-AP), 53BP1 (1:2,000, Abcam ab36823), α-tubulin (1:5,000, Sigma-Aldrich T6074).

### Drug treatment procedures

Where indicated, cells were treated with 0.4 mg/ml Nocodazole (Sigma-Aldrich M1404) for 16 hours, 10 μM MG132 for 3 hours, 2.5 μM MLN4924 for 3 hours, 100 μg/ml cycloheximide (CHX) for the indicated time.

### qRT-PCR

Total RNA was purified using RNeasy mini kits (Qiagen). cDNA was generated using Double Primed EcoDry kits (Takara). The qPCR reaction was carried out using PowerUp SYBR Green (Applied Biosystems) and the Applied Biosystems QuantStudio 3 Real-Time PCR system in a 96-well format. ROX was used as a reference dye for fluorescent signal normalization and for well-to-well optical variations correction. Bar graphs represent the relative ratios of target genes to β-actin housekeeping gene values. For each biological sample, triplicate reactions were analyzed using absolute relative quantification method alongside in-experiment standard curves for each primer set to control for primer efficiency. The oligos used for qRT-PCR analysis were: β-actin (F: 5’-CATGTACGTTGCTATCCAGGC-3’, R: 5’-CTCCTTAATGTCACGCACGAT-3’), GMCL1 (F: 5’-GGAGATTCCTGACCAGAACATTG-3’, R: 5’-CGACTGGGCTTTATCAAGACAT-3), GMCL2 (F: 5’-CCACGCAGCGGGTCTGT, R: 5’-TGGATTTTCTGGGTGACGATTATTT), p21 (F: 5’-TGTCCGTCAGAACCCATGC-3’, R: 5’-AAAGTCGAAGTTCCATCGCTC-3’), NOXA (F: 5’-CCAAGCCGTGACCAAGGAC-3’, R: 5’-CGCCACATTGTGTAGCACCT-3’), PUMA delta (F: 5’-GCCAGATTTGTGAGACAAGAGG-3’, R: 5’- CAGGCACCTAATTGGGCTC-3’).

### Immunoprecipitation, immunoblotting, and fractionation

For whole-cell lysates, cells were directly lysed in a buffer containing 25 mM Tris-HCl (pH 8.0), 150 mM NaCl, 0.2% NP-40, 10% glycerol, 1 mM EDTA, 1 mM EGTA, 2 mM MgCl_2_, 1 mM dithiothreitol (DTT), and 5 mM N-ethylmaleimide (Sigma-Aldrich). When cellular fractionation was performed, it followed a previously established method^36^. Briefly, cells were lysed in CSK buffer (10 mM PIPES, pH 7.0, 150 mM NaCl, 300 mM sucrose, 0.1% Triton X-100, 3 mM MgCl₂, 1 mM EGTA, 1 mM DTT, and 5 mM N-ethylmaleimide) for 5 minutes. The soluble fraction was collected by centrifugation at 3,000 × g for 3 minutes at 4°C. Cell pellets were subsequently washed in CSK buffer and then lysed in chromatin extraction buffer (50 mM Tris-HCl, pH 7.5, 150 mM NaCl, 0.2% NP-40, 10% glycerol, 1 mM EDTA, 1 mM EGTA, 4 mM MgCl_2_, 0.5 mM DTT, 5 mM N-ethylmaleimide, and 5 U/mL TurboNuclease (Accelagen)) for 30 minutes. Insoluble debris was removed by centrifugation at 20,000 × g for 15 minutes at 4°C. All buffers were supplemented with protease inhibitors (Complete ULTRA, Roche) and phosphatase inhibitors (PhosSTOP, Roche).

For immunoprecipitation and affinity purification, samples were incubated with FLAG-M2 magnetic beads (Sigma-Aldrich) at 4°C for 2 hours. Beads were thoroughly washed with lysis buffer, and protein elution was performed using either FLAG peptide (Sigma-Aldrich) for mass spectrometry analysis or 1x Laemmli sample buffer for Western Blot analysis.

### Immunoblotting

Western blotting was carried out as described previously^35^. Protein samples were resolved under denaturing and reducing conditions on 4%–12% Bis-Tris gels (NuPAGE) and transferred onto PVDF membranes (Immobilon-P, Millipore). Membranes were blocked with 5% nonfat dried milk, incubated overnight at 4°C with primary antibodies, followed by washes and incubation with HRP-conjugated secondary antibodies (Amersham GE). Immunoreactive bands were visualized using enhanced chemiluminescence reagents (Pierce) and detected with a ChemiDoc MP imaging system (Bio-Rad). Each Western blot experiment was conducted at least three times to ensure reproducibility, with representative blots shown in the figures.

### Cell cycle analysis by flow cytometry

BrdU incorporation and propidium iodide staining were performed following established protocols^37^. In brief, cells were pulse-labeled with 10 μM BrdU (Merck) for 45 minutes, followed by trypsinization and overnight fixation in ice-cold 70% ethanol. After washing with PBS and subsequently with PBST-BSA (1% BSA, 0.5% Tween-20 in PBS), DNA denaturation was carried out for 30 minutes using 2 N HCl with 0.5% Triton X-100. The cells were then washed in PBS and neutralized by resuspending them in 0.1 M sodium tetraborate (pH 8.5) for 2 minutes. To detect BrdU incorporation, cells were incubated for 1 hour at room temperature with FITC-conjugated anti-BrdU mouse antibodies (BD Transduction Laboratories, 556028) in PBST-BSA. After multiple washes, cells were resuspended in PBST-BSA containing 5 μg/mL propidium iodide (Invitrogen) and 20 μg/mL RNase A (Invitrogen). Flow cytometry analysis of cell cycle distribution was conducted using a CytoFlex Analyzer (Beckman Coulter) and data were processed with FlowJo v10 software (Becton Dickinson).

### Mass spectrometry analysis of GMCL1 immunoprecipitations

The eluted anti FLAG-tag antibody purified protein complexes were reduced with 2 μl of 0.2 M DTT for 1 h at 57 °C and subsequently alkylated with 2 μl of 0.5 M iodoacetamide (Sigma) for 45 min at room temperature in the dark. 250 ng of SP3 beads (Cytiva) were added proteins precipitated onto the beads by adding ethanol. Samples were placed in a thermomixer at 25°C for 10 min. Beads were washed three times with 80% ethanol and then digested overnight with 400ng of sequencing grade modified Trypsin (Promega) in 100 mM ammonium bicarbonate. Next, the samples were spun down 21,000 × g for 1 minute. The supernatant was transferred to a new tube while the beads were washed twice with 0.5% acetic acid. The washes were then combined with the supernatant collection. Samples were acidified with 10% TFA to pH1 and loaded onto a 0.1% TFA equilibrated Pierce C18 spin column using a microcentrifuge. The samples were rinsed twice with 0.1% TFA and twice more using 0.5% acetic acid. Peptides were eluted with 80% acetonitrile in 0.5% acetic acid. The organic solvent was removed using a SpeedVac concentrator and the sample reconstituted in 0.5% acetic acid.

An equal aliquot of each sample was loaded onto a trap column (Acclaim Pep-Map 100 precolumn, 75 μm × 2 cm, C18, 3 μm, 100 A, Thermo Scientific) connected to an analytical column (EASY-Spray column, 50 m × 75 μm internal diameter, PepMap RSLC C18, 2 μm, 100 Å, Thermo Scientific) using the autosampler of an Easy nLC 1200 (Thermo Fisher Scientific) with solvent A consisting of 2% acetonitrile in 0.5% acetic acid and solvent B consisting of 80% acetonitrile in 0.5% acetic acid. The peptide mixture was gradient eluted using the following gradient: 5% solvent B for 5 minutes, 5–35% solvent B in 60 min, 35–45% solvent B in 10 min, followed by 45–100% solvent B in 10 min. The samples were acquired on the Orbitrap Eclipse using the following parameters: full MS spectra resolution of 120,000, an AGC target of 4e5, maximum ion time of 50 ms, scan range from 400 to 1,500 m/z. The MS/MS spectra were collected with the following parameters: a resolution of 30,000, an AGC target of 2e5, maximum ion time of 30 ms, one microscan, 2 m/z isolation window, normalized collision energy (NCE) of 27 and a dynamic exclusion of 30 s. To identify binding partners, all acquired MS2 spectra were searched against a UniProt human database using Sequest HT within Proteome Discoverer 1.4 (Thermo Fisher Scientific). Fixed modifications were set on cysteine (carbamidomethyl), variable modifications of oxidation on methionine, and deamidation on glutamine and asparagine. The resulting peptide spectra matches and proteins are filtered to better than 1% false discovery rate (FDR) and only proteins with at least two different peptides are reported. Proteins differentially expressed between GMCL1 WT and EK, as determined by SAINT scores with a 5% FDR, were considered significantly enriched interactions when comparing to GMCL1 BTB.

### Taxol resistance analysis

Taxol resistance was analyzed across cancer cell lines catalogued in DepMap 24Q2. We integrated TP53 mutation status, *GMCL1* RNA and *TP53BP1* protein expression information across cells with the PRISM dataset^29^, to evaluate taxol resistance in the context of *GMCL1* or *TP53BP1* levels and *TP53* mutation.

### Cell viability and apoptosis assays

Cells (2500/well) were plated in a 96-well plate. The medium was replaced with 50 μL of medium containing the target siRNA (0.2 μL/well). After 24 hours, an additional 50 μL of the designated reagent was added. Luminescence was measured using BioTek Synergy Neo2 (Agilent) 48 hours post-treatment with CellTiter-Glo 2.0 Cell Viability Assay (Promega) or RealTime-Glo Annexin V Apoptosis and Necrosis Assay (Promega) according to the manufacturer’s protocol.

### Quantification and statistical analysis

Data analysis was performed using GraphPad Prism version 10.2.1. For comparisons involving three or more groups, One-way ANOVA followed by Bonferroni’s post hoc test or the Brown-Forsythe and Welch ANOVA test followed by Dunnett’S T3 multiple comparisons test was applied. For bioinformatic data analysis, two-group comparisons were performed using the pairwise Wilcoxon rank-sum test.

## Supplementary Material

1 Supplemental Figure 1. Mapping 53BP1 binding sites on GMCL1
(A) Predicted structure of GMCL1, domain architecture overview, and comparative analysis of the substrate-binding domain across Drosophila, fish, chicken, mouse, and human. Conserved amino acids are indicated by asterisks, with the human R433 residue highlighted in red.
(B) Schematic representation of 53BP1 domains.
(C) HEK293T cells were co-transfected with FLAG-GMCL1 and either EV, HA-53BP1 WT, or deletion mutants: HA-53BP1 ΔMFF, HA-53BP1 ΔN (lacking the N-terminus of MFF), HA-MFF, HA-MFF ΔOD (oligomerization domain), HA-MFF ΔGAR (glycine-arginine-rich motif), HA-MFF Δ1270–1484, or HA-MFF Δ1370–1484. Immunoprecipitation of 53BP1 was performed using HA beads, followed by immunoblotting of co-purified proteins.
(D) HEK293T cells were transfected with EV or FLAG-GMCL1, together with HA-53BP1 WT or mutants: HA-MFF, HA-53BP1 ΔMFF, HA-53BP1 ΔN (lacking the N-terminus of MFF), HA-53BP1 ΔTudor, and HA-53BP1 ΔC (lacking the C-terminus of MFF). 53BP1 was immunoprecipitated with HA-beads, followed by immunoblotting of co-purified proteins. Asterisk indicates non-specific bands.
(E) To narrow down the GMCL1-binding region on 53BP1, sequential 20-amino-acid deletions within the MFF domain were generated. HEK293T cells were co-transfected with EV or FLAG-GMCL1, along with HA-MFF, HA-MFF Δ1410–1430, and site-specific mutants (every three amino acids mutated within 1410–1430 region of 53BP1). Immunoprecipitation of 53BP1 was conducted with HA-beads, followed by immunoblotting.
(F) HEK293T cells were transfected with FLAG-GMCL1 or FLAG-GMCL2. GMCL1 and GMCL2 were immunoprecipitated with FLAG-beads and analyzed by immunoblotting.
Supplemental Figure 2. Mitotic stress imprints apoptotic memory in daughter cells
(A) RNA was extracted from FLAG-GMCL1-expressing U2OS cells (as in Figure 2B). p21 and NOXA mRNA levels were quantified by qPCR from three independent experiments. Error bars represent standard deviation.
(B) Stable GMCL1 KO U2OS cells were transduced with lentiviruses expressing EV, FLAG-GMCL1 WT, FLAG-GMCL1 EK, or FLAG-GMCL1 RA. Cells were synchronized in M phase, then released into fresh FBS-containing medium for 20 hours following shake-off. Daughter cells were fractionated into soluble and chromatin-bound fractions and analyzed by immunoblotting.
(C) RNA was extracted from same cells as in (B), and NOXA and PUMA mRNA levels were quantified by qPCR from three independent experiments. Error bars represent standard deviation.
(D) Cell cycle distribution was determined using BrdU pulse and PI staining of asynchronous U2OS WT or FLAG-GMCL1-expressing cells. Error bars represent standard deviation.
Supplemental Figure 3. Tissue-specific comparison of GMCL1 gene expression levels
The panel shows GMCL1 expression levels in normal cells and cancer cells based on GENT2^21^. The cells in the bottom table indicate where, GMCL1 expression levels were significantly higher in cancer cells (red), while the blue cells indicate cancers that were previously reported in the literature to exhibit Taxol resistance^13,38^.

## Data Availability

Original western blot images have been deposited at Mendeley at DOI:10.17632/gj3x6r263d.1 and are publicly available as of the date of publication. https://data.mendeley.com/preview/gj3x6r263d?a=1e452dfc-bf85-472c-83ce-0e997ba6fa40 The mass spectrometric raw files are accessible at https://massive.ucsd.edu under accession MassIVE MSV000097235 and at www.proteomexchange.org under accession PXD061458. Username: MSV000097235_reviewer, Password: TP53bdg-protein1
