# Supplementary material for: GMCL1 Controls 53BP1 Stability and Modulates Paclitaxel Sensitivity in Cancer
Source: bioRxiv. 2025 Mar 19:2025.03.18.643855. Preprint. [Version 1] doi: 10.1101/2025.03.18.643855 (PMC11957010; doi:10.1101/2025.03.18.643855)

Supplemental Figure1

bioRxiv preprint doi: https://doi.org/10.1101/2025.03.18.643855; this version posted March 20, 2025. The copyright holder for this preprint (which was not certified by peer review) is the author/funder, who has granted bioRxiv a license to display the preprint in perpetuity. It is made available under aCC-BY 4.0 International license.

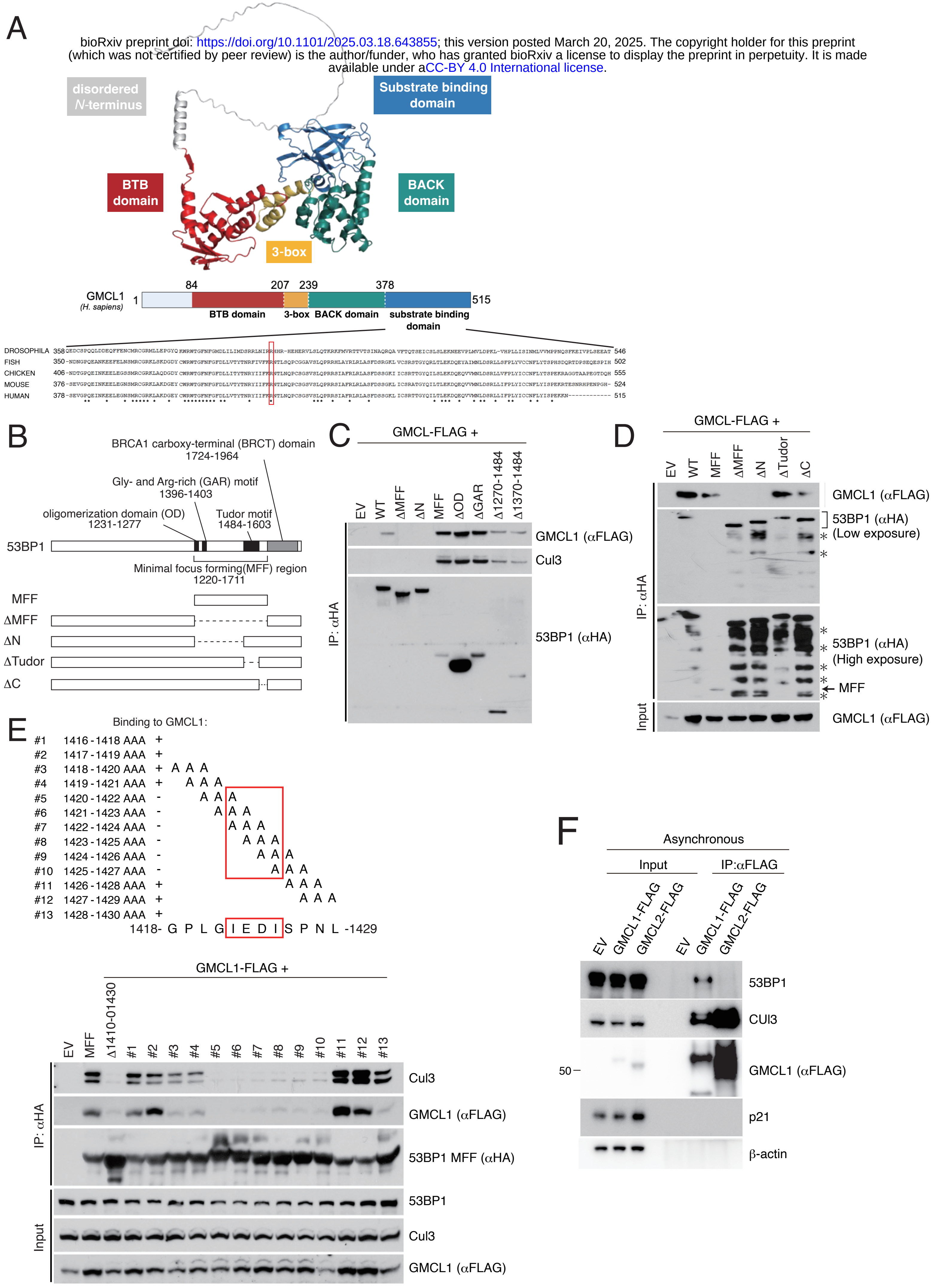

Supplemental Figure 2

bioRxiv preprint doi: https://doi.org/10.1101/2025.03.18.643855; this version posted March 20, 2025. The copyright holder for this preprint (which was not certified by peer review) is the author/funder, who has granted bioRxiv a license to display the preprint in perpetuity. It is made available under aCC-BY 4.0 International license.

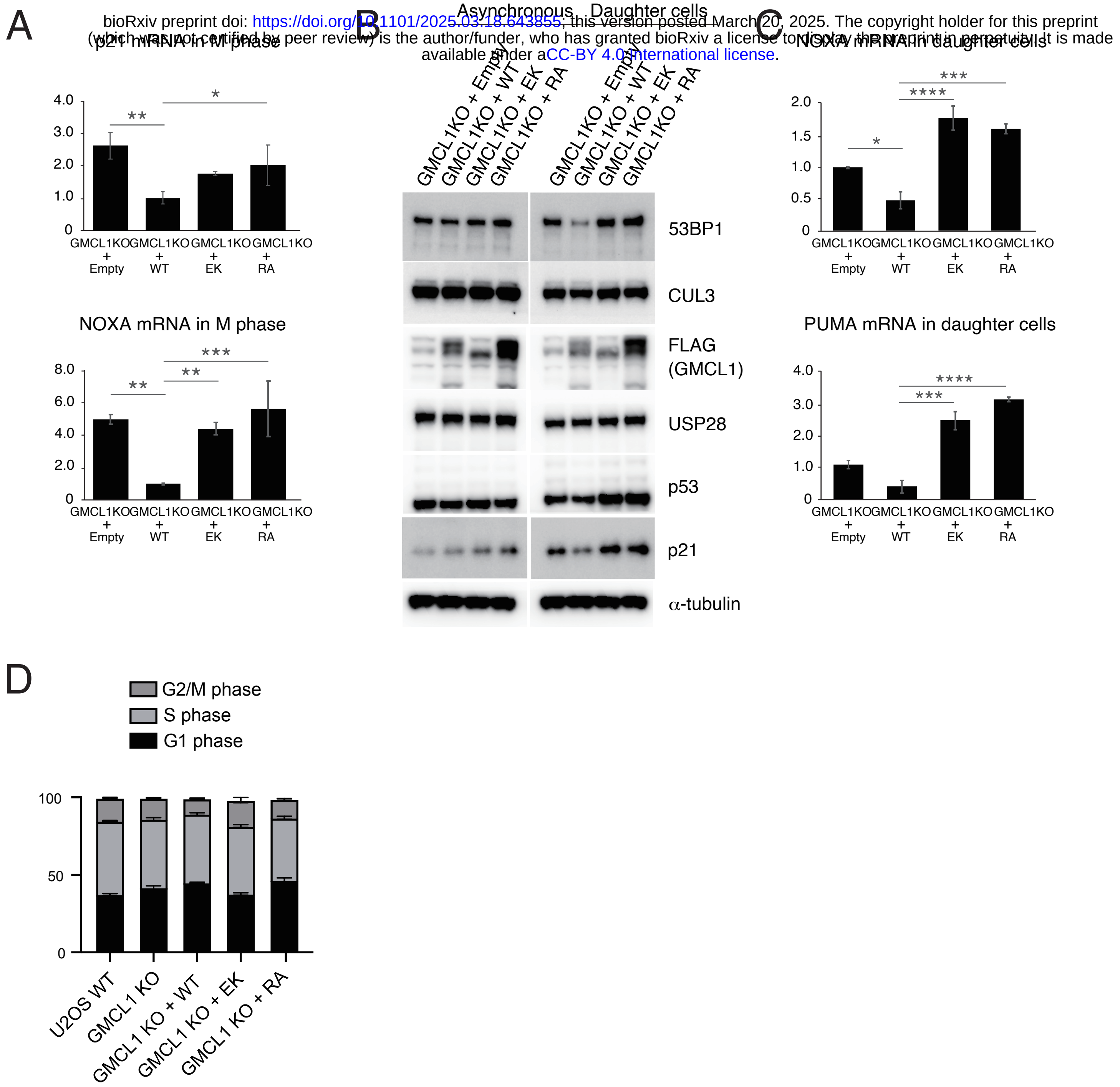

Supplemental Figure 3

bioRxiv preprint doi: https://doi.org/10.1101/2025.03.18.643855; this version posted March 20, 2025. The copyright holder for this preprint (which was not certified by peer review) is the author/funder, who has granted bioRxiv a license to display the preprint in perpetuity. It is made available under aCC-BY 4.0 International license.

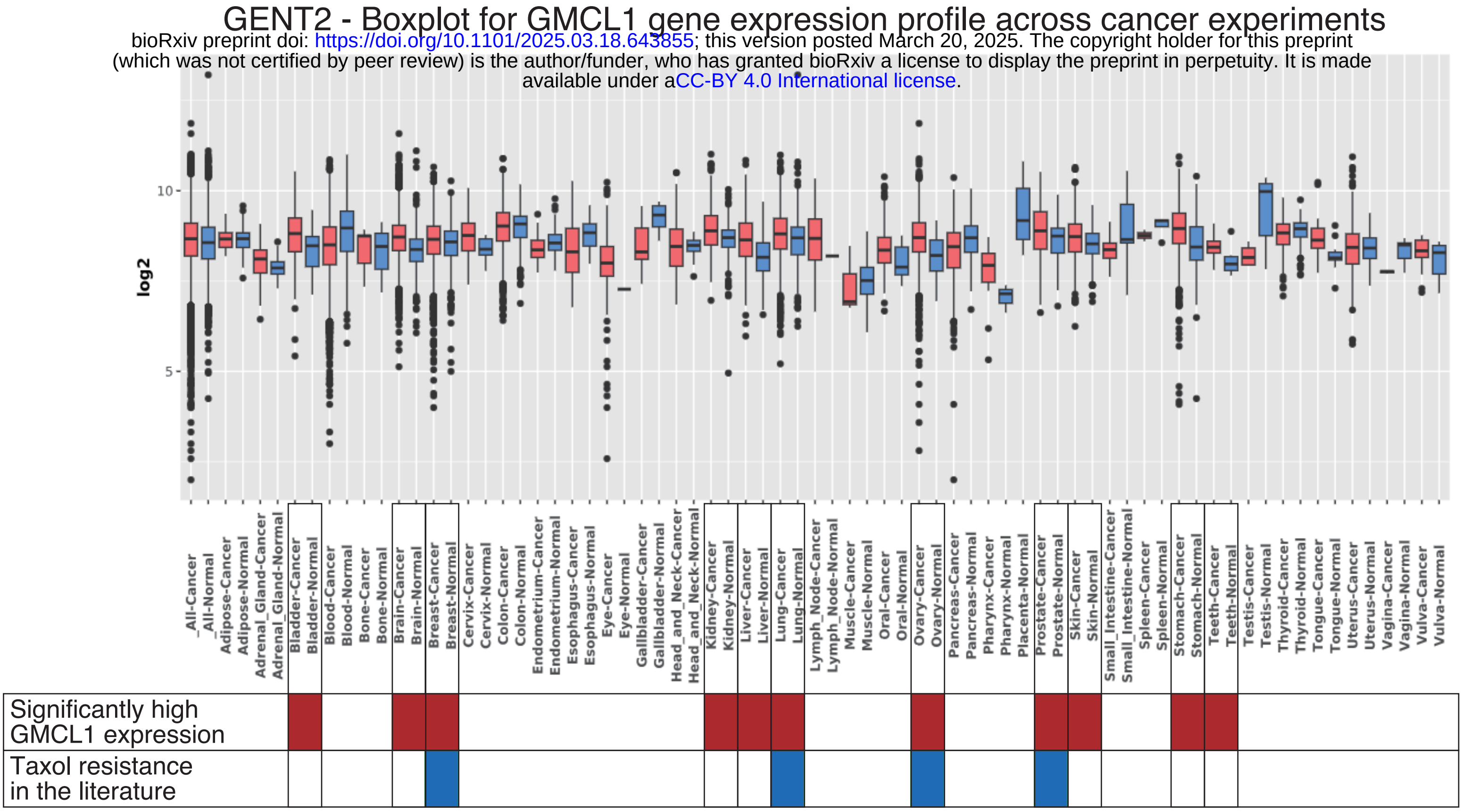

Supplement: 1 — Supplemental Figure 1. Mapping 53BP1 binding sites on GMCL1 (A) Predicted structure of GMCL1, domain architecture overview, and comparative analysis of the substrate-binding domain across Drosophila, fish, chicken, mouse, and human. Conserved amino acids are indicated by asterisks, with the human R433 residue highlighted in red. (B) Schematic representation of 53BP1 domains. (C) HEK293T cells were co-transfected with FLAG-GMCL1 and either EV, HA-53BP1 WT, or deletion mutants: HA-53BP1 ΔMFF, HA-53BP1 ΔN (lacking the N-terminus of MFF), HA-MFF, HA-MFF ΔOD (oligomerization domain), HA-MFF ΔGAR (glycine-arginine-rich motif), HA-MFF Δ1270–1484, or HA-MFF Δ1370–1484. Immunoprecipitation of 53BP1 was performed using HA beads, followed by immunoblotting of co-purified proteins. (D) HEK293T cells were transfected with EV or FLAG-GMCL1, together with HA-53BP1 WT or mutants: HA-MFF, HA-53BP1 ΔMFF, HA-53BP1 ΔN (lacking the N-terminus of MFF), HA-53BP1 ΔTudor, and HA-53BP1 ΔC (lacking the C-terminus of MFF). 53BP1 was immunoprecipitated with HA-beads, followed by immunoblotting of co-purified proteins. Asterisk indicates non-specific bands. (E) To narrow down the GMCL1-binding region on 53BP1, sequential 20-amino-acid deletions within the MFF domain were generated. HEK293T cells were co-transfected with EV or FLAG-GMCL1, along with HA-MFF, HA-MFF Δ1410–1430, and site-specific mutants (every three amino acids mutated within 1410–1430 region of 53BP1). Immunoprecipitation of 53BP1 was conducted with HA-beads, followed by immunoblotting. (F) HEK293T cells were transfected with FLAG-GMCL1 or FLAG-GMCL2. GMCL1 and GMCL2 were immunoprecipitated with FLAG-beads and analyzed by immunoblotting. Supplemental Figure 2. Mitotic stress imprints apoptotic memory in daughter cells (A) RNA was extracted from FLAG-GMCL1-expressing U2OS cells (as in Figure 2B). p21 and NOXA mRNA levels were quantified by qPCR from three independent experiments. Error bars represent standard deviation. (B) Stable [file NIHPP2025.03.18.643855V1-supplement-1.pdf]
